# Nonlinear encoding in diffractive information processing using linear optical materials

**DOI:** 10.1038/s41377-024-01529-8

**Published:** 2024-07-23

**Authors:** Yuhang Li, Jingxi Li, Aydogan Ozcan

**Affiliations:** 1 Electrical & Computer Engineering Department, University of California, Los Angeles, CA 90095 USA; 2 Bioengineering Department, University of California, Los Angeles, CA 90095 USA; 3 California NanoSystems Institute (CNSI), University of California, Los Angeles, CA 90095 USA

**Keywords:** Imaging and sensing, Optical techniques, Optical physics

## Abstract

Nonlinear encoding of optical information can be achieved using various forms of data representation. Here, we analyze the performances of different nonlinear information encoding strategies that can be employed in diffractive optical processors based on linear materials and shed light on their utility and performance gaps compared to the state-of-the-art digital deep neural networks. For a comprehensive evaluation, we used different datasets to compare the statistical inference performance of simpler-to-implement nonlinear encoding strategies that involve, e.g., phase encoding, against data repetition-based nonlinear encoding strategies. We show that data repetition within a diffractive volume (e.g., through an optical cavity or cascaded introduction of the input data) causes the loss of the universal linear transformation capability of a diffractive optical processor. Therefore, data repetition-based diffractive blocks cannot provide optical analogs to fully connected or convolutional layers commonly employed in digital neural networks. However, they can still be effectively trained for specific inference tasks and achieve enhanced accuracy, benefiting from the nonlinear encoding of the input information. Our results also reveal that phase encoding of input information without data repetition provides a simpler nonlinear encoding strategy with comparable statistical inference accuracy to data repetition-based diffractive processors. Our analyses and conclusions would be of broad interest to explore the push-pull relationship between linear material-based diffractive optical systems and nonlinear encoding strategies in visual information processors.

## Introduction

Optical nonlinear materials^1–9^ can be used to generate nonlinear responses in an optical information processing system to potentially improve its approximation power for various computing functions acting on, e.g., 2D or 3D visual information of a scene. However, most existing nonlinear optical materials require high-power illumination due to their weak nonlinear coefficients or suffer from low speed and/or optical losses. Therefore, linear optical materials can provide relatively simpler alternatives for building and designing optical visual processors—albeit at the cost of performance limitations. A visual processor formed by linear optical materials can, in fact, benefit from nonlinear information encoding strategies to improve its inference accuracy for various computational tasks, all the way from arbitrary function approximation to data classification. Arguably, one of the simplest forms of nonlinear encoding of information for a linear optical system (without nonlinear materials) is phase encoding, implemented, for example, through a simple spatial light modulator (SLM) *or* the use of phase-only objects, where the visual information of interest, $i({\rm{x}},{\rm{y}})$ is encoded in the phase channel of a wave: ${e}^{{\rm{j}}i\left({\rm{x}},{\rm{y}}\right)}$. For any linear optical processor ($L$), we, in general, have:

1 $$L\left\{{e}^{{\rm{j}}[{i}_{1}\left({\rm{x}},{\rm{y}}\right)\,+\,{i}_{2}({\rm{x}},{\rm{y}})]}\right\}\,\ne\, L\left\{{e}^{{\rm{j}}{i}_{1}\left({\rm{x}},{\rm{y}}\right)}\right\}+L\left\{{e}^{{\rm{j}}{i}_{2}\left({\rm{x}},{\rm{y}}\right)}\right\}$$

This nonlinear encoding of optical information in the phase channel of a coherent diffractive processor has been successfully used to approximate nonlinear functions using linear diffractive optical systems for various applications, including image classification, quantitative phase imaging (QPI), phase-to-intensity transformations and image encryption, among others^10–19^.

Another recently emerging, exciting form of nonlinear optical information encoding strategy involves virtual data repetition within a linear material-based diffractive volume^20,21^. In these systems, virtual copies of the information of interest create cascaded secondary waves that interfere with each other through a linear material system (e.g., based on a cavity^21^ and/or SLM^20^), without using nonlinear optical materials. This creates a form of nonlinear information encoding since virtual copies of $i$ diffract and interact with $i$ in a cascaded manner, creating optical waves that have a nonlinear dependency on $i$.

Here, we analyze the performances of different nonlinear information encoding strategies implemented using optical diffraction in linear material systems and shed light on their utility and limitations. Our analyses reveal that data repetition-based nonlinear encoding is equivalent to *input-dependent* spatially varying point spread function (PSF) engineering between the input and output apertures of a diffractive linear system. Therefore, such data-repeating diffractive structures cannot perform arbitrarily selected fully connected or convolutional layers commonly used in digital neural networks. Despite this architectural feature that breaks the universal linear transformation capability of diffractive computing, data repetition within a diffractive processor boosts its inference accuracy, benefiting from the nonlinear information encoding. Through simulations, we compared the statistical inference accuracy of (1) data repetition-based task-optimized diffractive processors and (2) phase-encoding-based simpler-to-implement diffractive optical processors by performing image classification using different datasets (e.g., MNIST^22^, Fashion-MNIST^23^, and CIFAR-10^24^). Our findings show that despite its simplicity, phase-encoding-based nonlinear encoding (without data repetition) can generally deliver inference accuracies statistically comparable to the data repetition-based diffractive computing methods.

Furthermore, diffractive optical processors without data repetition do not need pre-processing of input information through a digital system, which is required for visual data repetition; this can be time-consuming, especially for phase-only input objects due to the need for digital phase recovery and pre-processing before data repetition in a trainable architecture can take place. However, data repetition-based nonlinear encoding approaches still present some unique merits; notably, data-repeating diffractive networks might possess advantages in terms of noise resilience^20^, a desired feature for noisy scenarios and related sensing applications.

We should also note that the free-space diffractive optical processors analyzed in this work, with and without data repetition, should not be confused with some of the recent coupled mode-based neuromorphic computing architectures^25^ that encode and process information using, e.g., the frequencies and coupling rates of various modes, which might provide a better fit for integrated photonics implementations, outside the scope of this article. Furthermore, the mathematical meaning and implications of data repetition^25^ in such coupled mode-based neuromorphic processors are different from the visual data repetition considered here in diffractive optical processors. Regardless, for any input information that is represented in e.g., the phase and/or amplitude of a scene/object, such coupled mode-based neuromorphic computing architectures would also require pre-processing of input optical information through a digital system before any data repetition can physically occur.

Our results and analyses provided in this manuscript are of broad interest to design diffractive optical information processors using linear optical materials involving various nonlinear encoding strategies to further improve our understanding of visual computing using engineered light-matter interactions.

## Results

### Impact of data repetition in a diffractive optical processor

**Fig. 1:**
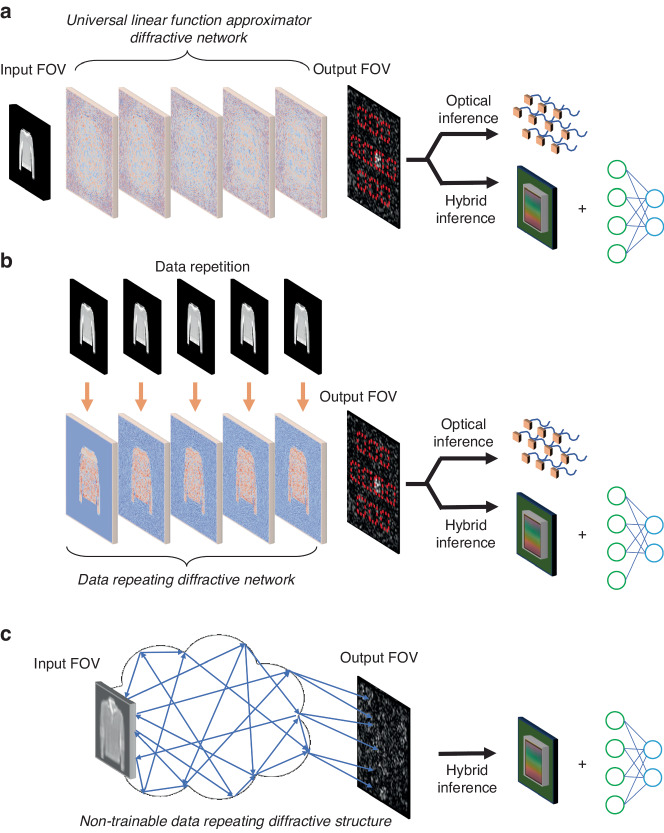
Different nonlinear encoding methods for diffractive optical processors using linear optical materials. **a** A schematic illustration of a universal linear function approximator diffractive network, which is composed of trainable transmissive and/or reflective diffractive layers. The input optical information in this method is fed into the diffractive processing system solely at the input FOV aperture. **b** A schematic representation of data repetition-based diffractive processors with trainable transmissive and/or reflective layers, where the input data are fed into the phase modulation functions of the successive diffractive data repetition layers, resulting in input-dependent spatially varying PSFs between the input and output apertures. Both of the diffractive processor architectures shown in (**a**, **b**) are capable of performing inference all-optically or in a hybrid manner (involving collaboration with a jointly trained electronic neural network at the backend). **c** The concept of non-trainable virtual data-repeating diffractive structures, which require integration with a trainable electronic neural network backend (shown on the right) for statistical inference

**Fig. 2:**
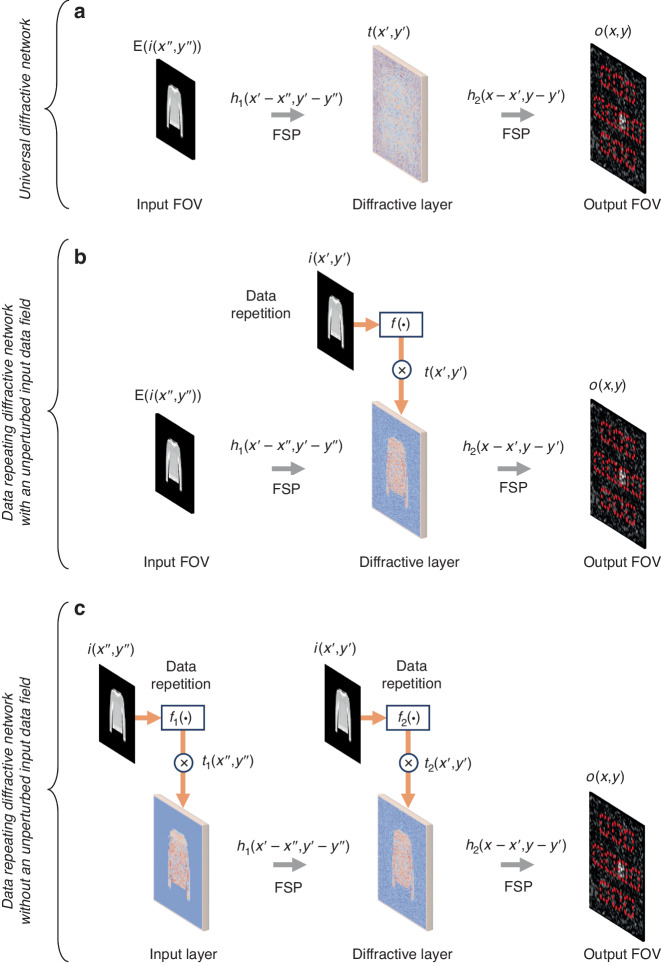
Schematic illustration used for analyzing the impact of data repetition in a trainable diffractive optical processor. **a** Schematic for a diffractive optical processor without data repetition, which can be used as a universal linear transformer between its input and output FOVs. ${\rm{E}}\left(\cdot \right)$ is the input data encoding function, which can be nonlinear. **b**, **c** Schematic for a trainable diffractive optical processor using data repetition. Compared to (**b**), data repetition also occurs at the input plane in (**c**). $f\left(\cdot \right)$ is a trainable encoding function at a given data repetition layer; $t\left(\cdot \right)$ is a trainable diffractive transmission/reflection function. FSP free-space propagation

A trainable coherent diffractive optical processor (Fig. 1a) composed of successive transmissive (and/or reflective) surfaces that are spatially structured using linear materials forms a universal linear transformer that can approximate any complex-valued linear transformation between its input and output fields-of-view (FOVs)^26–30^. Such diffractive optical processors can also be optimized for spatially and temporally incoherent illumination, preserving their universality for linear function approximation under spatially incoherent and/or broadband illumination^29,30^. Without loss of generality, here we consider a trainable diffractive optical processor illuminated with spatially coherent and monochromatic light at a wavelength of $\lambda$. Using, for example, deep learning-based optimization, such a diffractive visual processor can perform an arbitrary set of complex-valued spatially varying PSFs between its input and output apertures. Considering a single trainable diffractive layer (see Fig. 2a) with an optimized 2D transmission function of $t\left(x^{\prime} ,y^{\prime} \right)$, the output optical field ($o$) in response to a 2D input field that encodes input data $(i)$ through an input data encoding function ${\rm{E}}\left(\cdot \right)$ can be written as:

2 $$\begin{array}{ll}o\left(x,y\right)=\sum\limits _{{x}^{{\prime} }{y}^{{\prime} }}\sum\limits _{{x}^{{\prime} {\prime} }{y}^{{\prime} {\prime} }}{\rm{E}}(i\left({x}^{{\prime} {\prime} },{y}^{{\prime} {\prime} }\right))\cdot {h}_{1}\left({x}^{{\prime} }-{x}^{{\prime} {\prime} },{y}^{{\prime} }-{y}^{{\prime} {\prime} }\right)\cdot t\left({x}^{{\prime} },{y}^{{\prime} }\right)\cdot {h}_{2}\left(x-{x}^{{\prime} },y-{y}^{{\prime} }\right)\\\qquad\qquad =\sum\limits _{{x}^{{\prime} {\prime} }{y}^{{\prime} {\prime} }}{\rm{E}}(i\left({x}^{{\prime} {\prime} },{y}^{{\prime} {\prime} }\right))\cdot h\left({x}^{{\prime} {\prime} },{y}^{{\prime} {\prime} },x,y\right)\end{array}$$

where, ${h}_{1}$ and ${h}_{2}$ refer to the free-space propagation functions that connect the input plane (${x}^{{\prime} {\prime} },{y}^{{\prime} {\prime} }$) to the diffractive layer plane (${x}^{{\prime} },{y}^{{\prime} }$), and the diffractive layer plane (${x}^{{\prime} },{y}^{{\prime} }$) to the output plane ($x,y$), respectively (see Fig. 2a). Based on this diffractive architecture, any arbitrary complex-valued linear transformation from the input FOV to the output FOV can be performed (at the diffraction limit of light) by optimizing $t\left({x}^{\prime} ,{y}^{\prime} \right)$, assuming that there is a sufficiently large number of independent degrees of freedom in $t\left({x}^{\prime} ,{y}^{\prime} \right)$—larger than or equal to ${N}_{i}\times {N}_{o}$, where ${N}_{i}$ and ${N}_{o}$ refer to the space-bandwidth product of the input and output apertures of the diffractive processor, respectively^26–30^. By accordingly optimizing the transmission function, $t\left({x}^{\prime} ,{y}^{\prime} \right)$, any arbitrary desired set of spatially varying complex-valued PSFs (i.e., $h\left({x}^{{\prime} {\prime} },{y}^{{\prime} {\prime} },x,y\right)$) between the input and output FOVs can be approximated^26,27^:

3 $$h\left({x}^{{\prime} {\prime} },{y}^{{\prime} {\prime} },x,y\right)=\sum\limits _{{x}^{{\prime} }{y}^{{\prime} }}t\left({x}^{{\prime} },{y}^{{\prime} }\right)\cdot {h}_{1}\left({x}^{{\prime} }-{x}^{{\prime} {\prime} },{y}^{{\prime} }-{y}^{{\prime} {\prime} }\right)\cdot {h}_{2}\left(x-{x}^{{\prime} },y-{y}^{{\prime} }\right)$$

As a universal linear transformer, an optimized diffractive optical processor can perform fully connected complex-valued linear transformations between its input and output apertures. Needless to say, it can also perform any arbitrarily selected complex-valued convolution operation between its input and output planes.

These above conclusions hold even if the input data encoding function ${\rm{E}}\left(\cdot \right)$ is nonlinear such as ${\rm{E}}\left(i\right)={e}^{{\rm{j}}i\left({\rm{x}},{\rm{y}}\right)}$ which represents phase-only input objects or data.

On the other hand, Fig. 1b depicts an alternative scheme that uses the repetition of input data within the diffractive volume. This scheme, however, breaks the universal linear function approximation capability of diffractive computing. To see this, assume that the input data $i$ is virtually repeated on the plane of $t\left({x}^{{\prime} },{y}^{{\prime} }\right)$ through a certain encoding function $f\left(\cdot \right)$, which is, in general, a trainable/optimizable function (Fig. 2b). As a result of this virtual data repetition, the output field can be written as:

4 $$\begin{array}{ll}o\left(x,y\right)\,{\boldsymbol{=}}\,\sum\limits _{{x}^{{\prime} }{y}^{{\prime} }}\sum\limits _{{x}^{{\prime} {\prime} }{y}^{{\prime} {\prime} }}{\rm{E}}\left(i\left({x}^{{\prime} {\prime} },{y}^{{\prime} {\prime} }\right)\right)\cdot {h}_{1}\left({x}^{{\prime} }-{x}^{{\prime} {\prime} },{y}^{{\prime} }-{y}^{{\prime} {\prime} }\right)\cdot t\left({x}^{{\prime} },{y}^{{\prime} }\right)\cdot f\left(i\left({x}^{{\prime} },{y}^{{\prime} }\right)\right)\cdot {h}_{2}(x-{x}^{{\prime} },y-{y}^{{\prime} })\\\qquad\qquad {\boldsymbol{=}}\sum\limits _{{x}^{{\prime} {\prime} }{y}^{{\prime} {\prime} }}{\rm{E}}\left(i\left({x}^{{\prime} {\prime} },{y}^{{\prime} {\prime} }\right)\right)\mathop{\sum}\limits_{{x}^{{\prime} }{y}^{{\prime} }}t\left({x}^{{\prime} },{y}^{{\prime} }\right)\cdot f\left(i\left({x}^{{\prime} },{y}^{{\prime} }\right)\right)\cdot {h}_{1}({x}^{{\prime} }-{x}^{{\prime} {\prime} },{y}^{{\prime} }-{y}^{{\prime} {\prime} })\cdot {h}_{2}\left(x-{x}^{{\prime} },y-{y}^{{\prime} }\right)\end{array}$$

where the term $\sum _{{x}^{{\prime} }{y}^{{\prime} }}t({x}^{{\prime} },{y}^{{\prime} })\cdot f\left(i\left({x}^{{\prime} },{y}^{{\prime} }\right)\right)\,{\cdot}\, {h}_{1}({x}^{{\prime} }-{x}^{{\prime} {\prime} },{y}^{{\prime} }-{y}^{{\prime} {\prime} })\cdot {h}_{2}(x-{x}^{{\prime} },y-{y}^{{\prime} })$ represents a set of *input-dependent spatially varying PSFs* between the input (${x}^{{\prime} {\prime} },{y}^{{\prime} {\prime} }$) and output ($x,y$) apertures. This means, under data repetition, a diffractive optical processor cannot perform an arbitrarily selected fully connected or convolutional transformation between the input and output apertures since its function will change for each input.

The same conclusions also apply to the diffractive architectures, where optimizable data repetition also occurs at the input plane^20^, introducing two different trainable encoding functions ${f}_{1}\left(\cdot \right)$ and ${f}_{2}\left(\cdot \right)$, for $\left({x}^{{\prime} {\prime} },{y}^{{\prime} {\prime} }\right)$ and $\left({x}^{{\prime} },{y}^{{\prime} }\right)$ planes, respectively (see Fig. 2c). For this alternative configuration of data repetition-based diffractive processor, the output field can be expressed as:

5 $$\begin{array}{ll}o\left(x,y\right){\boldsymbol{=}}\sum\limits _{{x}^{{\prime} }{y}^{{\prime} }}\sum\limits _{{x}^{{\prime} {\prime} }{y}^{{\prime} {\prime} }}{t}_{1}\left({x}^{{\prime} {\prime} },{y}^{{\prime} {\prime} }\right)\cdot {f}_{1}\left(i\left({x}^{{\prime} {\prime} },{y}^{{\prime} {\prime} }\right)\right)\cdot {h}_{1}\left({x}^{{\prime} }-{x}^{{\prime} {\prime} },{y}^{{\prime} }-{y}^{{\prime} {\prime} }\right)\cdot {t}_{2}\left({x}^{{\prime} },{y}^{{\prime} }\right)\cdot {f}_{2}\left(i\left({x}^{{\prime} },{y}^{{\prime} }\right)\right)\cdot {h}_{2}(x-{x}^{{\prime} },y-{y}^{{\prime} })\\\qquad\qquad {\boldsymbol{=}}\sum\limits _{{x}^{{\prime} {\prime} }{y}^{{\prime} {\prime} }}{\rm{E}}\left(i\left({x}^{{\prime} {\prime} },{y}^{{\prime} {\prime} }\right)\right)\cdot \mathop{\sum}\limits _{{x}^{{\prime} }{y}^{{\prime} }}{t}_{2}\left({x}^{{\prime} },{y}^{{\prime} }\right)\cdot {f}_{2}\left(i\left({x}^{{\prime} },{y}^{{\prime} }\right)\right)\cdot {h}_{1}({x}^{{\prime} }-{x}^{{\prime} {\prime} },{y}^{{\prime} }-{y}^{{\prime} {\prime} })\cdot {h}_{2}\left(x-{x}^{{\prime} },y-{y}^{{\prime} }\right)\end{array}$$

where ${t}_{1}\left(\cdot \right)$ and ${t}_{2}\left(\cdot \right)$ represent the optimizable transmission functions at $\left({x}^{{\prime} {\prime} },{y}^{{\prime} {\prime} }\right)$ and $\left({x}^{{\prime} },{y}^{{\prime} }\right)$ planes, respectively, and ${\rm{E}}\left(i\left({x}^{{\prime} {\prime} },{y}^{{\prime} {\prime} }\right)\right)\,{\boldsymbol{=}}\,{t}_{1}\left({x}^{{\prime} {\prime} },{y}^{{\prime} {\prime} }\right)\cdot {f}_{1}\left(i\left({x}^{{\prime} {\prime} },{y}^{{\prime} {\prime} }\right)\right)$ (see Fig. 2c). For example, for the data repeating architecture of ref. ^20^, we have ${f}_{1}\left(i\left({x}^{{\prime} {\prime} },{y}^{{\prime} {\prime} }\right)\right)\,{\boldsymbol{=}}\,{e}^{j2\pi {S}_{1}\left({x}^{{\prime} {\prime} },{y}^{{\prime} {\prime} }\right)i\left({x}^{{\prime} {\prime} },{y}^{{\prime} {\prime} }\right)}$, ${f}_{2}\left(i\left({x}^{{\prime} },{y}^{{\prime} }\right)\right)={e}^{j2\pi {S}_{2}\left({x}^{{\prime} },{y}^{{\prime} }\right)i\left({x}^{{\prime} },{y}^{{\prime} }\right)}$, ${t}_{1}\left({x}^{{\prime} {\prime} },{y}^{{\prime} {\prime} }\right)={e}^{j2\pi {B}_{1}\left({x}^{{\prime} {\prime} },{y}^{{\prime} {\prime} }\right)}$ and ${t}_{2}\left({x}^{{\prime} },{y}^{{\prime} }\right)={e}^{j2\pi {B}_{2}\left({x}^{{\prime} },{y}^{{\prime} }\right)}$, which were implemented digitally using an SLM. $S\left(\cdot \right)$ and $B\left(\cdot \right)$ are optimizable parameters at each data-repeating layer for applying scaling and bias to the input data, respectively^20^.

Similar to Eq. (4), this new Eq. (5) also reveals a set of *input-dependent spatially varying PSFs* between the input and output apertures, breaking the universal linear function approximation capability of diffractive computing.

The above-discussed conclusions also apply to a cavity-based diffractive design, where multiple virtual copies of the input information ${\rm{E}}\left(i\left({x}^{{\prime} {\prime} },{y}^{{\prime} {\prime} }\right)\right)$ create secondary waves cascaded to each other, as illustrated by Fig. 1c. In fact, any optical medium formed by a set of partially reflective and/or transmissive surfaces in 3D can be used to create virtual replicas of the input information to create cascaded representations with nonlinear encoding, where the diffracted/scattered copies of the input interact with itself in a cascaded and iterative manner. However, a cavity with an arbitrary 3D topology presents a complicated physical process, which makes the data repetition-related encoding function $f\left(\cdot \right)$ much more complex. Even if custom modulation of the optical waves inside a cavity could be implemented through e.g., controllable elements, this kind of design still cannot exhibit universality for performing an arbitrary linear transformation between the input and output FOVs due to the existence of the input-dependent spatially varying PSFs resulting from $f\left(i\right)$.

In all of these cases, the input data (or their diffracted and/or scattered versions) are virtually repeated within a diffractive linear optical system (e.g., Figs. 1b, c and 2b, c); this cascaded repetition of the input data brings nonlinear encoding to the optical processor at the cost of losing universal linear transformation capability. *Therefore, data-repeating diffractive blocks cannot provide all-optical analogs to convolutional or fully connected layers commonly employed in digital neural networks.*

Note that this input-dependent functional representation due to data repetition within the diffractive optical volume should not be confused with some of the skip connections or related digital neural network architectures^31,32^ that feed input data structures at deeper parts of an electronic neural network. Regardless of the depth and specific arrangement of their data blocks, all these computing architectures fundamentally rely on fully connected and/or convolutional linear blocks, the coefficients of which are input-*independent*. Hence, the inherent architecture of data repetition-based diffractive processors does not support the implementation of these fundamental building blocks performing fully connected or convolutional filters.

On the other hand, data repetition-based diffractive processors can be intuitively perceived as a highly simplified optical analog of the *dynamic convolution kernel* concept used in some neural network architectures^33–38^. In these dynamic digital neural network modules, the weights of the convolution kernels are dynamically generated based on the input data/images, and they can spatially vary across different image regions. Such digital adaptability can reduce the model size compared to standard CNNs (convolutional neural networks) that achieve a similar level of inference performance. In these dynamic architectures, convolutional kernels are typically determined by processing the input data through either an additional neural network^33^ or specialized digital processing units with linear convolutional filters followed by nonlinear activation functions^34,35^. In this context, a data repetition-based diffractive processor can be viewed as a highly simplified version of the same concept where the dynamic kernel generation only employs a scaling function $S\left(\cdot \right)$ together with an additive bias term $B\left(\cdot \right)$. Therefore, despite the lack of universality, nonlinear encoding through data repetition blocks, if trained appropriately, can provide inference accuracy advantages for task-specific diffractive processors. In the next sub-section, we will evaluate the statistical inference performance of such nonlinear encoding schemes and compare them against some of the simpler-to-perform nonlinear schemes, such as e.g., phase encoding ${\rm{E}}\left(i\right)={e}^{{\rm{j}}i\left({\rm{x}},{\rm{y}}\right)}$ which represents phase-only input objects or data.

As emphasized in our introduction, the data-repeating diffractive optical processors considered here should not be confused with data repetition in a coupled mode-based neuromorphic architecture^25^. In the former case of diffractive optical processors, the input data encoding function ${\rm{E}}\left(i\right)$ is an optical wave that fills the input aperture of our system; for example, for a phase-only input object of interest, we have an optical field ${\rm{E}}\left(i\right)={e}^{{\rm{j}}i\left({\rm{x}},{\rm{y}}\right)}$ that directly represents the object/scene information through the phase channel of the wave, introduced at the input FOV of the visual optical processor. However, in the latter case of a coupled mode-based neuromorphic processor, the input information to be processed is not represented as an optical wave at the input FOV, making it more suitable for integrated photonics-based implementations that can represent the input information and trainable parameters of the system through various mode frequencies and coupling rates. Using a probe beam that fills in, for example, the input waveguide, it could deliver energy to the output waveguide or detector through a trained/optimized coupled mode architecture which can approximate a nonlinear function at the output. However, such an optimized function approximator architecture based on coupled modes does not constitute a visual optical processor since the probe/input beam does not carry the information of a scene or object of interest, and therefore, the input–output visual information processing relationships described in Eqs. (2–5) do not hold. In fact, the input probe beam in this case simply serves as the source of energy for computing, as opposed to carrying the object/scene information through an optical wave.

### Performance comparison of different nonlinear encoding schemes

As depicted in Fig. 1a, we selected a diffractive optical processor with five phase-only diffractive layers as our testbed to perform the classification of input images. Within this configuration, each diffractive layer is spatially coded with 200 × 200 diffractive features, with each feature having a lateral size of ~λ/2, capable of performing full phase modulation ranging from 0 to 2π. The input plane, the diffractive layers and the output plane are sequentially arranged along the optical axis with a spacing of 40$\lambda$, resulting in a diffractive volume that axially spans 240$\lambda$. Figure 1a illustrates a universal diffractive optical network architecture, where the information from the input data $\left(i\right)$ is fed into the processor *solely* at the input FOV based on a certain encoding function, ${\rm{E}}\left(i\right)$. Here, we consider/compare three different encoding methods at the input FOV: (1) amplitude encoding, (2) intensity encoding, and (3) phase encoding. Specifically, for a given input data $i$, the input coherent field, ${\rm{E}}(i)=|A|{e}^{j2\pi \phi }$, that encodes information of $i$ using these different encoding strategies can be expressed as:

1. Amplitude encoding:

6 $${\rm{E}}\left(i\right)=\left|A\right|=i$$

(2) Intensity encoding:

7 $${\rm{E}}\left(i\right)=\left|A\right|=\sqrt{i}$$

(3) Phase encoding:

8 $${\rm{E}}\left(i\right)={e}^{j2\pi \phi }={e}^{j{\rm{\alpha }}\pi i}$$

where ${\alpha }$ is a phase-encoding constant. For the datasets that we used in this work, $\left|i\right|=i$ and therefore the amplitude encoding here is linear, while the other two (intensity encoding and phase encoding) represent the nonlinear encoding of the input data. Without loss of generality, we used ${\alpha }=1$ in our analysis. After being processed by the diffractive optical system, the resulting optical fields form an intensity pattern at the output FOV, which is measured/sampled by multiple detectors to produce the output class signals. More details regarding the optical forward model of diffractive networks can be found in “Methods”.

Different from the architecture of Fig. 1a, the diffractive optical processor shown in Fig. 1b further employs an encoding method based on data repetition^20^. Its distinction from the method shown in Fig. 1a is that the input image is not fed through the input FOV but into the phase features of the diffractive layers, driving their phase modulations to be dependent on the input data. In this case, the phase modulation parameter for each diffractive feature, $\phi$, can be written as^20^

9 $$\phi =\left({\rm{Padding}}\left\{i\right\}\times S+B\right)$$

where $S$ and $B$ are trainable parameters, representing the scaling and bias factors, respectively—as defined earlier. ${\rm{Padding}}\{\cdot \}$ denotes a zero-padding operation, which is used to ensure that the resulting size of $\phi$ can match that of the data-repeating diffractive layers. Once the training is completed, $S$ and $B$ will be fixed and remain unchanged during the inference (blind testing) stage. Since $i$ represents the input data information, $\phi$ signifies an input-dependent phase modulation, which requires the digital implementation of the parameters $S$ and $B$ connected to all the diffractive features (see “Methods” for more details). For this data repetition-based diffractive architecture, we only consider using phase encoding at the input FOV to facilitate a direct comparison with the universal diffractive networks using phase encoding. Therefore, for a given data repetition diffractive layer *k* we have: ${f}_{k}\left(i\right)={e}^{j2\pi {S}_{k}i}$, and ${t}_{k}={e}^{j2\pi {B}_{k}}$ (see e.g., Fig. 2c).

At the output plane of a given diffractive processor, we consider the use of two distinct detection schemes: conventional detection and *differential* detection. The conventional detection scheme^10^ refers to using one distinct detector at the output plane for each data class (i.e., 10 detectors for MNIST, Fashion-MNIST or CIFAR-10 datasets). Each of these detectors integrates the measured light intensity within its detection area, and produces a class score corresponding to one of the data classes. The maximum score obtained among all these 10 detectors indicates the predicted class label. The second detection scheme, i.e., differential detection, was introduced in our previous work^11^. Its difference from the conventional scheme is that each data class of interest is assigned to a pair of “positive” and “negative” detectors, where the differential normalized signal for each pair serves as the inference score for that class, virtually introducing positive and negative numbers to the detection plane. Therefore, the differential detection scheme would use, e.g., 10 × 2 = 20 detectors at the output FOV for MNIST, Fashion-MNIST or CIFAR-10 datasets (see “Methods” for details).

**Fig. 3:**
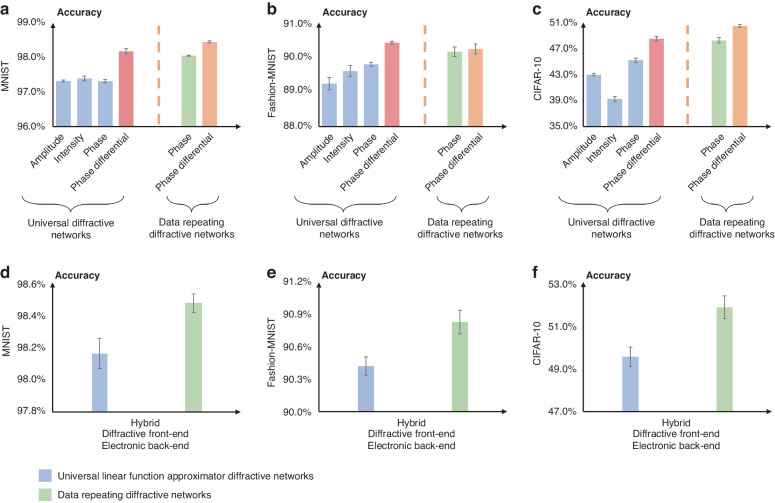
Performance comparison of different nonlinear encoding schemes for trainable diffractive optical processors. **a**–**c** Comparison of the blind testing accuracies for various diffractive optical processors that perform optical inference of (**a**) MNIST handwritten digits, **b** Fashion-MNIST images, and **c** CIFAR-10 images. Different information encoding strategies, including amplitude, intensity, and phase encoding, are used in the universal linear function approximator diffractive networks. A differential detection scheme is also considered for both the universal diffractive networks and data-repeating diffractive networks. **d**–**f** Comparison of the blind testing classification accuracies for optoelectronic hybrid diffractive optical processors with and without data repetition. The hybrid diffractive processor here includes a jointly trained diffractive optical front end and a one-layer fully connected electronic network backend

Based on these configurations introduced above, we trained various diffractive network models using the training images from three datasets: MNIST handwritten digits^22^, Fashion-MNIST^23^, and CIFAR-10^24^. For each configuration and dataset combination, we trained *N* = 5 different diffractive models to obtain statistically interpretable results; the differences in these five models primarily stem from distinct sequences of feeding the training data. After the training, we numerically blind-tested each model using the test images of these datasets (see “Methods” for details). The blind test results corresponding to the MNIST, Fashion-MNIST and CIFAR-10 datasets are summarized in Fig. 3a–c, respectively.

From these results, we have the following important observations: for the universal diffractive optical processor structure without input data repetition, we found out that the use of amplitude, intensity, and phase-encoding methods (Eqs. (6–8)) produced very similar blind test accuracies on the MNIST dataset, with classification accuracies of 97.32 ± 0.07%, 97.39 ± 0.03% and 97.31 ± 0.05%, respectively. For the Fashion-MNIST dataset, however, these classification accuracies present a slightly larger variation, reported as 89.24 ± 0.16%, 89.60 ± 0.18%, and 89.80 ± 0.06%, for the amplitude, intensity, and phase-encoding methods, respectively. Among these, the diffractive models using the phase encoding (${\rm{E}}\left(i\right)={e}^{j\pi i}$) outperformed the others, with a mean classification accuracy improvement of ~0.5% and ~0.2% compared to those using the amplitude and intensity encoding, respectively. As for the CIFAR-10 dataset, the classification accuracies are 42.97 ± 0.19%, 39.25 ± 0.42% and 45.19 ± 0.31%, for the amplitude, intensity, and phase-encoding methods, respectively. The phase encoding demonstrated an improvement of ~2.2% and ~5.9% compared to the amplitude and intensity encoding methods, respectively.

In fact, a much bigger performance jump in inference accuracy is observed when the differential detection scheme^11^ is employed for the phase-encoded diffractive networks without data repetition: we achieved blind test accuracies of 98.18 ± 0.08%, 90.42 ± 0.04% and 48.53 ± 0.32% for the MNIST, Fashion-MNIST, and CIFAR-10 datasets, respectively (see the red bars in Fig. 3a–c). These provide statistically significant improvements (with *P* values of <0.05) with respect to the amplitude, intensity, and phase-encoding results (blue bars) provided in Fig. 3a–c. This inference improvement can be attributed to the expanded coverage of the differential detection scheme in which all real numbers (as opposed to non-negative numbers that represent intensity values) are included by employing 2× more output detectors, thereby enhancing the generalization power of a diffractive optical processor.

In comparison to these, the data repetition-based phase-encoded diffractive processors reported with green bars in Fig. 3a–c provide blind testing accuracies of 98.05 ± 0.01%, 90.16 ± 0.14% and 48.29 ± 0.42% for the MNIST, Fashion-MNIST and CIFAR-10 datasets, respectively. These comparative analyses indicate that for the MNIST dataset, there is a statistically significant improvement of ~0.13% mean accuracy (*P* value = 0.033) in the blind inference performance of a differential diffractive network without data repetition (red bar in Fig. 3a) compared to a data repetition-based diffractive processor (green bar in Fig. 3a). The situation is similar for the Fashion-MNIST dataset (Fig. 3b) which reveals that there is a statistically significant improvement of ~0.26% mean accuracy (*P* value of <0.05) in the blind inference performance of a differential phase-encoded diffractive network without data repetition compared to a data repetition-based diffractive processor (green bar in Fig. 3b). However, for the CIFAR-10 dataset (Fig. 3c), there is no statistically significant difference between the two models (*P* value = 0.39). Overall, these indicate relatively small performance differences between the two architectures, although, on average, a differential diffractive network without data repetition appears to be more accurate in its inference. In these comparisons, each architecture’s total number of trainable diffractive features remained the same (see “Methods”).

The same figures also compare the performances of the differential detection scheme applied to data repetition-based diffractive networks, shown with the orange bars in Fig. 3a–c. These comparisons reveal that the differential detection scheme provides a statistically significant improvement of ~0.4% and ~2.19% mean accuracy (*P* values < 0.05) in the MNIST and CIFAR-10 blind inference performance of a data repetition-based diffractive processor compared to its non-differential counterpart, respectively (see the orange and green bars in Fig. 3a, c). However, we observed no statistically significant difference for the Fashion-MNIST blind inference performance of a data repetition-based diffractive processor compared to its differential counterpart (see the green and orange bars in Fig. 3b).

These statistical analyses reveal that data repetition-based diffractive processors are indeed successful in their inference performances despite the loss of their universal linear function approximation power; however, they are statistically on par with the corresponding performances of phase-encoded differential diffractive networks without data repetition. These results confirm that the use of nonlinear encoding through ${\rm{E}}\left(i\right)$ and/or $f\left(i\right)$ in a diffractive processor represents an effective architecture that can bring a certain degree of performance enhancement in data classification tasks. However, data repetition-based diffractive processor architectures arguably present more complex optical systems compared to e.g., differential diffractive networks without data repetition, which in general perform comparably to the inference results of data repetition-based nonlinear encoding networks, as shown in Fig. 3a–c.

Regarding architectural complexity, the data repetition strategy, by design, necessitates digitization and pre-processing of the unknown input information through an imaging system, which is not needed for standard diffractive processors without data repetition. For example, if the input of interest is a phase-only object, i.e., ${\rm{E}}\left(i\right)={e}^{{\rm{j}}i\left({\rm{x}},{\rm{y}}\right)}$, the data repetition-based diffractive processors would need first to perform phase retrieval using e.g., holographic imaging systems, before the phase data can be digitally processed/scaled and replicated within the diffractive volume. On the contrary, standard diffractive optical processors without data repetition can directly act on such phase-only input object data without any pre-processing or digital phase recovery steps.

Next, we further explored the performance comparison of different nonlinear encoding methods in the case of optoelectronic hybrid computing, where the diffractive optical processor forms the front-end and an electronic/digital network forms the jointly trained backend, processing the output of the diffractive system (see Fig. 1 and “Methods” for details). An earlier demonstration of this synergy between diffractive processors and electronic networks revealed that hybrid diffractive computing can achieve enhanced image classification performance compared to their all-optical counterparts, while the data size for electronic network inputs can also be substantially compressed^12^, allowing for low pixel count imagers and reduced complexity in digital network structures. For the investigation of hybrid systems in this paper, we used an array of 4 × 4 = 16 detectors at the output aperture, which are digitally fed into a backend electronic processing unit, with a fully connected digital neural network. After their joint training with the same image datasets and optimization approach, the results are reported in Fig. 3d–f. The blind testing results on the MNIST dataset achieved classification accuracies of 98.47 ± 0.06% and 98.16 ± 0.09% with and without the data repetition; these accuracies became 90.83 ± 0.11% and 90.42 ± 0.09%, respectively, when using the Fashion-MNIST dataset; and 51.83 ± 0.53% and 49.54% ± 0.45%, respectively, when using the CIFAR-10 dataset. These results reveal statistically significant mean accuracy improvements of ~0.3%, ~0.4%, and ~2.3% for the MNIST, Fashion-MNIST and CIFAR-10 datasets, respectively.

**Fig. 4:**
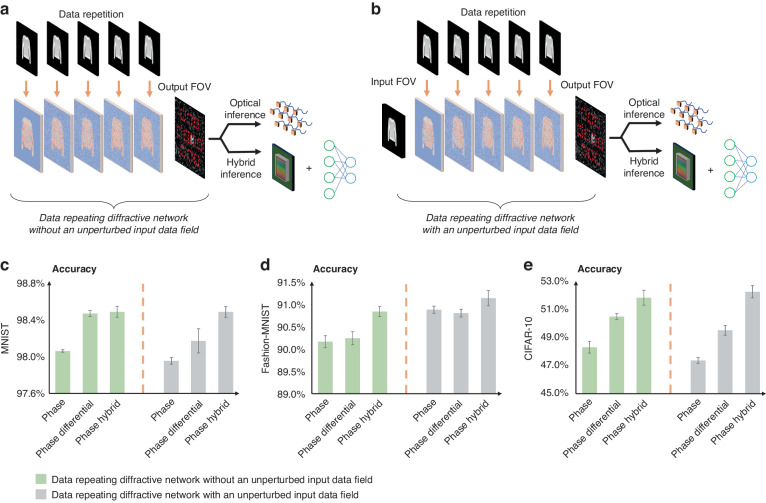
Performance comparison of data-repeating diffractive optical processors with and without an unperturbed input data field. **a** Schematic for a data-repeating diffractive optical processor without an unperturbed input data field at the input FOV^20^. **b** Schematic for a data-repeating diffractive optical processor with an unperturbed input data field. Note that configurations (**a**, **b**) have an identical number of trainable diffractive degrees of freedom, which is equal to 5 × 200 × 200 = 200 K. **c**–**e** Comparison of the blind testing classification accuracies for the data-repeating diffractive optical processors with and without an unprocessed phase-encoded input data field at the input aperture. This comparison is performed based on three different data repetition-based diffractive processor configurations: (1) using the conventional detection scheme with 10 output detectors, (2) using the differential detection scheme with 20 output detectors, and (3) using a jointly trained diffractive optical front end and a one-layer fully connected electronic network backend, which is referred to as “hybrid”

In the data-repeating diffractive processor designs reported so far (e.g., Fig. 4a), the input data were always subject to an encoding function and then fed into various data-repeating diffractive layers, following the architecture of ref. 20. In other words, the input information from the object was never presented to the diffractive processor in an unperturbed form. In this work, we also devised an alternative design for the data repetition-based diffraction processor designs, which optically feeds the unperturbed data into the diffractive volume without any digital nonlinear encoding, as illustrated in Fig. 4b. We hypothesized that this alternative data repetition strategy would perform enhanced statistical inference benefiting from the unperturbed information content, encoded at its input, i.e., ${\rm{E}}\left(i\right)$—see Fig. 4b.

Figure 4d–f reports the inference results for these two different data repetition strategies (Fig. 4a, b) within a diffractive processor. When using the conventional and differential detection schemes, as shown in Fig. 4c, the data-repeating diffractive processor designs with an *unperturbed* input achieved MNIST blind testing accuracies of 97.95 ± 0.04% and 98.16 ± 0.13% (differential), respectively. These indicate statistically significant decreases of ~0.1% and ~0.3% in the mean classification accuracies compared to the performances of their counterpart designs without an unperturbed input. A similar situation is observed with the CIFAR-10 dataset, as depicted in Fig. 4e, where the data-repeating diffractive processor designs with an unperturbed input achieved accuracies of 47.36 ± 0.21% and 49.51 ± 0.36% (differential), showing statistically significant decreases of ~0.9% in both cases. However, the situation is reversed when using the Fashion-MNIST dataset, as shown in Fig. 4d. The data-repeating diffractive processor designs with an unperturbed input achieved accuracies of 90.88 ± 0.08% and 90.80 ± 0.09% using the conventional and differential detection schemes, respectively - demonstrating statistically significant improvements of ~0.7% and ~0.6% in the classification accuracies compared to the performances of their counterpart designs without an unperturbed input. Moreover, we performed a comparison for the hybrid diffractive processor architecture that combines a jointly trained data-repeating diffractive optical front end and a one-layer fully connected electronic network backend. Here, the data-repeating diffractive processor designs with an unperturbed input achieved MNIST, Fashion-MNIST, and CIFAR-10 blind testing accuracies of 98.47 ± 0.06%, 91.13 ± 0.17% and 52.25% ± 0.43%, respectively. These results indicate a statistically significant improvement of ~0.3% in the mean classification accuracy for the Fashion-MNIST dataset when compared to the performance of the counterpart hybrid design shown in Fig. 4a. As for the MNIST and CIFAR-10 dataset performances shown in Fig. 4c, e, there were no statistically significant differences between the classification accuracies of the two approaches. These findings reveal that using an unperturbed input within a data-repeating diffractive processor depicted in Fig. 4b offers certain performance benefits (especially for the Fashion-MNIST dataset), although the statistical improvements are marginal.

## Discussion

In this manuscript, we demonstrated that phase encoding of input information provides a straightforward and simple-to-implement nonlinear encoding strategy in diffractive processor architectures with comparable statistical inference results to data repetition-based diffractive architectures. Our analyses also reveal that data repetition within a diffractive volume causes the loss of the universal linear transformation capability of a diffractive processor; stated differently, data repetition-based diffractive blocks cannot provide optical analogs to fully connected or convolutional blocks employed in digital networks. Furthermore, phase encoding enables a universal diffractive network to directly process the optical information corresponding to phase-only objects in an all-optical manner—i.e., without the need for digital phase retrieval and pre-processing of information (which are required for data repetition-based trainable designs). Direct access to and processing of optical phase information of input objects not only streamlines the information processing pipeline but also enhances the efficiency and speed of optical inference. Our analyses also confirmed that data repetition-based diffractive architectures could bolster the diffractive system’s capability in task-specific image classification tasks. This enhancement stems from their ability to improve the inference accuracy through the repetitive introduction of the input data. As we pointed out earlier, these data repetition-based diffractive processors can be considered, at least intuitively, as a simplified optical analog of the dynamic convolution kernel concept used in the literature. Additionally, data repetition-based architectures can be used to demonstrate noise resilience, a desired feature in noisy scenarios and related sensing applications, by leveraging information redundancy for increased precision.

It is worth noting that while we primarily explored and compared different nonlinear encoding strategies in this study, we did not consider the involvement of optical nonlinear materials acting as activation layers. Future research could benefit from integrating diffractive processors with various forms of optical nonlinearity and evaluating the resulting performance improvements. In addition, as demonstrated in this study, hybrid systems (with a diffractive front end and a digital backend) generally outperform the all-optical solutions in their inference performance. Therefore, developing innovative structures together with more effective methods to jointly train such hybrid systems could better harness the unique advantages of each component—optical and digital—potentially leading to enhanced performance and robustness.

Furthermore, many experimental factors, such as fabrication errors and physical misalignments, can affect the performance of diffractive processors during the experimental deployment stage. Investigating the inherent robustness of different nonlinear encoding strategies to such imperfections, as well as their integration with vaccination-based training strategies^39^ or in situ training methods^40^, would provide more comprehensive guidance on the implementation and limitations of these approaches. These considerations would be crucial for future research and practical implementations of diffractive optical processors.

Throughout the manuscript, our analyses assumed that diffractive optical processors consist of several stacked diffractive layers interconnected through free-space light propagation, as commonly used in the literature^10,13,41,42^. Our forward model employs the angular spectrum method for light propagation, a broadly applicable technique known for its accuracy, covering all the propagating modes in free space. While our forward model does not account for multiple reflections between the diffractive layers, it is important to note that such cascaded reflections are much weaker than the transmitted light and, thus, have a negligible impact on the optimization process. This simplification does not compromise the model’s experimental validity since a given diffractive model also acts as a 3D filter for such undesired secondary sources that were ignored in the optimization process; stated differently, a by-product of the entire optimization process is that the resulting diffractive layers collectively filter out some of these undesired sources of secondary reflections, scattering them outside the output FOV. The foundation of our model has been extensively validated through various experiments^10,11,16,18,43^, providing a good match to the corresponding numerical model in each case, further supporting the accuracy of our forward model and diffractive processor design scheme.

Finally, our numerical analyses were conducted using coherent monochromatic light, which has many practical, real-world applications such as holographic microscopy and sensing, laser-based imaging systems, optical communications, and biomedical imaging. These applications, and many others, benefit from the precise control of the wave information carried by coherent light. In addition to coherent illumination, diffractive optical processors can also be designed to accommodate temporally and spatially incoherent illumination. By optimizing the layers for multiple wavelengths of illumination, a diffractive processor can be effectively designed to operate under broadband illumination conditions^18,19,29,43–47^. Similarly, by incorporating spatial incoherence into the forward model simulations, we can design diffractive processors that function effectively with spatially incoherent illumination^30,48^. Without loss of generality, our current study focuses on coherent monochromatic light to establish a foundational understanding of nonlinear encoding strategies in diffractive information processing using linear optical materials by leveraging the precise control that coherent processors offer. Future work could explore the extension of these principles to spatially or temporally incoherent illumination scenarios, further broadening the applicability of diffractive optical processors in practical settings.

## Methods

### Numerical forward model of a diffractive optical processor

The architecture of a diffractive optical network comprises a series of diffractive layers, transmissive and/or reflective, which collectively modulate the incoming wavefronts of the object. In our numerical forward model, these diffractive layers are treated as thin, planar elements that passively modulate the incoming optical fields. The transmission coefficient ${t}^{(l)}$ at a specific spatial coordinate (${x}_{i},{y}_{i},{z}_{i}$) for the *l*th diffractive layer, can be expressed as:

10 $${t}^{(l)}\left({x}_{i},{y}_{i},{z}_{i}\right)={a}^{(l)}\left({x}_{i},{y}_{i},{z}_{i}\right)\exp \left(j{\phi }^{(l)}\left({x}_{i},{y}_{i},{z}_{i}\right)\right)$$

where $a$ and $\phi$ denote the amplitude and phase coefficients, respectively. The optical fields at successive diffractive layers are connected through free-space propagation, which can be mathematically modeled using the Rayleigh–Sommerfeld diffraction equation, as described by the following equation:

11 $${w}_{i}^{(l)}\left(x,y,z\right)=\frac{z-{z}_{i}}{{r}^{2}}\left(\frac{1}{2\pi r}+\frac{1}{j\lambda }\right)\exp \left(\frac{j2\pi r}{\lambda }\right)$$

Here, ${w}_{i}^{(l)}\left(x,y,z\right)$ is the complex field at the ${i}^{\text{th}}$ pixel of the ${l}^{\text{th}}$ layer with a spatial coordinate of $\left(x,y,z\right)$, which can be interpreted as a secondary wave generated from the source at $\left({x}_{i},{y}_{i},{z}_{i}\right)$. The propagation distance $r$ is given by $\sqrt{{(x-{x}_{i})}^{2}+{(y-{y}_{i})}^{2}+{(z-{z}_{i})}^{2}}$ and $j$ equals $\sqrt{-1}$.

For the *l*th layer (where $l$ ≥ 1, with the input plane treated as the 0th layer), the modulated optical field ${u}^{(l)}$ at location (${x}_{i},{y}_{i},{z}_{i}$) can be calculated as:

12 $${u}^{(l)}\left({x}_{i},{y}_{i},{z}_{i}\right)={t}^{(l)}\left({x}_{i},{y}_{i},{z}_{i}\right)\,\cdot\, \sum\limits _{k\in K}{u}^{(l-1)}\left({x}_{k},{y}_{k},{z}_{k}\right)\,\cdot\, {w}_{k}^{(l-1)}\left({x}_{i},{y}_{i},{z}_{i}\right)$$

where $K$ encompasses all the diffractive features on the preceding layer. In our numerical implementations, the angular spectrum method^10^ was utilized to compute Eq. (12), which can be rewritten as follows:

13 $${u}^{(l)}\left({x}_{i},{y}_{i},{z}_{i}\right)={{\mathcal{F}}}^{-1}\left\{{\mathcal{F}}\left\{{u}^{(l-1)}\left({x}_{k},{y}_{k},{z}_{k}\right)\right\}\cdot H\left({f}_{x},{f}_{y},\,{z}_{i}-{z}_{k}\right)\right\}$$

where ${\mathcal{F}}$ and ${{\mathcal{F}}}^{-1}$ denote the two-dimensional Fourier transform and its inverse, respectively, both computed via a fast Fourier transform (FFT). The transfer function of free space, $H({f}_{x},{f}_{y},{z}_{i}-{z}_{k})$, is defined as:

14 $$H\left({f}_{x},{f}_{y},\,{z}_{i}-{z}_{k}\right)=\left\{\begin{array}{ll}\exp \left\{j\frac{2\pi \left({z}_{i}\,-\,{z}_{k}\right)}{\lambda }\sqrt{1-{\left(\lambda {f}_{x}\right)}^{2}-{\left(\lambda {f}_{y}\right)}^{2}}\right\}\qquad,{f}_{x}^{2}+{f}_{y}^{2}\, < \frac{1}{{\lambda }^{2}}\\ 0,\,{f}_{x}^{2}+{f}_{y}^{2}\ge \displaystyle\frac{1}{{\lambda }^{2}}\end{array}\right.$$

where ${f}_{x}$ and ${f}_{y}$ correspond to the spatial frequencies in the *x* and *y* directions, respectively.

### Data preparation

The classification performances of all the diffractive models discussed in this work were evaluated using three benchmark datasets: MNIST^22^, Fashion-MNIST^23^, and CIFAR-10^24^. For the MNIST and Fashion-MNIST datasets, 55,000 samples constituted the training set, while the remaining 15,000 images were allocated into two separate sets, containing 5000 and 10,000 images for validation and blind testing purposes, respectively. For the CIFAR-10 dataset, 50,000 samples were partitioned into training and validation sets, comprising 40,000 and 10,000 images, respectively. An additional 10,000 samples formed the testing set.

### Training loss functions

In standard diffractive optical networks without the differential detection strategy, the class score for a specific data class $c$, denoted as ${s}_{c}^{({\rm{Stan}}.)}$, is calculated using the formula:

15 $${s}_{c}^{({Stan}.)}=\frac{1}{T}\cdot \frac{{s}_{c}}{\max \left({s}_{c}\right)}$$

where, ${s}_{c}$ denotes the optical signal corresponding to the detector allocated to class $c$. $\max \left({s}_{c}\right)$ denotes the maximum optical signal across all the detectors. $T$ is a non-trainable hyperparameter that is also known as the “temperature” parameter^49^ in machine learning literature. It is used only during the training phase to expedite convergence and enhance the model’s accuracy. In our implementation, the value of $T$ was empirically set to 0.1.

For the diffractive models employing the differential detection scheme in image classification tasks, each data class is assigned to a pair of detectors at the output plane: one for collecting “positive” class signals and another for acquiring “negative” class signals. The differential class scores, ${s}_{c}^{({\rm{Diff}}.)}$, are calculated using^11^:

16 $${s}_{c}^{({\rm{Diff}}.)}=\frac{1}{T}\cdot \frac{{s}_{c,+}-{s}_{c,-}}{{s}_{c,+}+{s}_{c,-}}$$

where ${s}_{c,+}$ and ${s}_{c,-}$ denote the positive and negative signals for the ${c}^{\text{th}}$ class, respectively. The hyperparameter $T$ was only used during the training stage to accelerate the convergence of the models, and was empirically chosen as 0.1.

### Other implementation details

The minimum spatial sampling interval designated for the optical field simulations was set to ~0.53λ, identical to the lateral size of each diffractive feature on the diffractive layers. The input FOV spans 64λ × 64λ, accommodating input data. The shape and size of the output detectors were fixed, and we assumed each of them was a square with a width of 6.4λ. For the diffractive network models jointly trained with a digital processing backend using a single fully connected layer, the output intensity pattern of the diffractive network is measured at the output plane within a region of 64λ × 64λ (corresponding to 120 × 120 pixels in sampled fields). This is followed by an average pooling operation with a binning factor of 30, shrinking the output dimension from 120 × 120 to 4 × 4. This tensor of 4 × 4 is further flattened to be a vector and used as the input to a fully connected layer, which ultimately produces an output tensor consisting of 10 elements, representing the class scores for the 10 data classes in the dataset.

In our diffractive neural networks, each diffractive layer has 200 × 200 diffractive features that perform phase-only modulation of complex fields. For the data repetition diffractive networks, since the input object $i$ has a size of 64$\lambda$ × 64$\lambda$ (corresponding to 120 × 120 pixels) that is smaller than that of the diffractive layers 106.7$\lambda$ × 106.7$\lambda$ (corresponding to 200 × 200 pixels), we used a zero-padding operation ${\rm{Padding}}\{\cdot \}$ with 21.3$\lambda$ (corresponding to 40 pixels) on each side to match the resulting size of $\phi$ with that of the diffractive layers, as indicated by Eq. (9). Consequently, the phase modulation values $\phi$ of the diffractive features located in the zero-padded area are solely dictated by the trainable bias function $B$.

In this work, all the diffractive network models and fully connected layers were trained using the PyTorch framework (v1.11.0, Meta Platforms Inc.). The optimizer employed was an Adam optimizer^50,51^, with its default parameter settings in PyTorch identically applied across all models. In the training process, a batch size of 128 was used. The learning rate was initially set as 0.001. Each diffractive model underwent a total of 150 training epochs. Computational resources for the training comprised a workstation equipped with a Nvidia GeForce GTX 1080Ti graphical processing unit (GPU), an Intel Core i7 8700 central processing unit (CPU), and 64 GB of random-access memory (RAM), operating on the Windows 10 platform (Microsoft Inc.). The average time for training a diffractive optical network model is ~3 h.

## Data Availability

All the data and methods needed to evaluate the conclusions of this work are presented in the main text. Additional data can be requested from the corresponding author. The codes used in this work use standard libraries and scripts that are publicly available in PyTorch.
